# Integrative machine learning and gene regulatory network analysis identifies novel genes for leafy head formation in *Brassica rapa* and *B. oleracea*

**DOI:** 10.1186/s43897-026-00230-1

**Published:** 2026-06-08

**Authors:** Wei Sun, Jian Wu, Xu Cai, Yue Chen, Aalt D. J. van Dijk, Xiaowu Wang, Guusje Bonnema

**Affiliations:** 1 State Key Laboratory of Vegetable Biobreeding, Institute of Vegetables and Flowers, Chinese Academy of Agricultural Sciences, Beijing, 100081 China; 2 Plant Breeding, Wageningen University and Research, 6708 PB Wageningen, The Netherlands; 3 Bioinformatics Group, Wageningen University and Research, 6708 PB Wageningen, The Netherlands; 4 Biosystems Data Analysis, Swammerdam Institute for Life Sciences, University of Amsterdam, 1090 GE Amsterdam, The Netherlands

**Keywords:** Leafy head, Machine learning, Gene regulatory network, *Brassica rapa*, *Brassica oleracea*

## Abstract

Leafy head formation is a crucial developmental process in *Brassica* crops. Here, an integrative approach combining machine learning and gene regulatory network analysis was employed to identify novel genes involved in leafy head formation in Chinese cabbage (*Brassica rapa)* and cabbage (*Brassica oleracea)*. Random Forest models, trained with 47 known leafy head-related genes, demonstrated robust performance with mean AUC values of 0.87 and 0.85 for *B. rapa* and *B. oleracea*, respectively. By filtering the model predictions, we identified 11 genes predicted with high confidence which were shared between both species. To further reveal the regulatory mechanisms, we constructed gene regulatory networks for genes in both species. By integrating ML predictions with these networks, we identified key regulatory clusters specifically related to leafy head formation. Network centrality analysis revealed many core genes in key clusters, including important transcription factors such as *ANT, GRF2, GRF3,* and *TCX3*, suggesting crucial roles in leafy head formation across the two species. The parallel detection of the same genes and similar network structures in the two species supports the validity of our findings. Our integrative approach provides novel insights into the genetic regulation of leafy head formation and sets the stage for future functional studies of *Brassica* species.

**Supplementary Information:**

The online version contains supplementary material available at 10.1186/s43897-026-00230-1.

## Core

We have developed an integrative approach combining machine learning and gene regulatory network analysis to identify novel genes involved in leafy head formation in *Brassica rapa* and *Brassica oleracea.* Random Forest models demonstrated robust performance in predicting new candidates, while network analysis revealed key regulatory clusters and important transcription factors. The parallel detection of similar genes and network structures in both species mutually validates our findings, providing novel insights into the genetic regulation of leafy head formation for *Brassica* crops.

## Gene & accession numbers

Gene & accession numbers information can be found in supplementary tables.

## Introduction

Leafy head formation is an important developmental process in *Brassica* species, such as *Brassica rapa* and *Brassica oleracea*, which include economically important vegetables like Chinese cabbage and cabbage. A well-shaped leafy head determines the marketability and consumer preference of these crops.

The formation of leafy heads in *Brassica* species is regulated by a complex network of interacting genes. To elucidate the molecular mechanism, advanced methods such as genome mapping, transcriptome and miRNA expression profiling have been used, and several related genes have been identified. Cheng et al. (2016) completed whole-genome resequencing of *B. rapa* and *B. oleracea*, identifying six key genes and revealing that leafy head formation involves phytohormones, signal transduction pathways, and leaf adaxial-abaxial polarity. Among these factors, auxins have emerged as central regulators, as first demonstrated by Ito and Kato (1957) who showed that auxin concentration and distribution influence leaf curvature in *B. rapa*. Subsequent studies by He et al. (2000), Gao et al. (2017), Li et al. (2019) have confirmed auxin's crucial role and identified many auxin-associated candidate genes involved in heading. Additionally, genes controlling leaf polarity, particularly in the YABBY and HD-ZIP III families, are essential for establishing the adaxial-abaxial asymmetry required for proper leaf curvature (Manuela and Xu 2020). Despite these advances in identifying individual genes and pathways, the complete regulatory network governing leafy head formation remains unclear, particularly how these diverse components integrate to control this complex developmental process.

Recent advancements in high-throughput sequencing technologies have generated large amounts of genomic and transcriptomic data for *Brassica* species (Guo et al. 2021; S. Li et al. 2021a, b, c; Yuan et al. 2021; Wang et al. 2023; Cai et al. 2024). These datasets provide a valuable resource for investigating the molecular basis of complex traits like leafy head formation. However, the huge amount and complexity of these data bring substantial challenges for traditional analytical approaches. To address these challenges, two powerful tools have emerged: machine learning (ML) algorithms and Gene Regulatory Network (GRN) analysis. ML offers several advantages for studying leafy head formation in *Brassica* species. Firstly, ML algorithms can efficiently handle high-dimensional data and identify patterns and correlations between several types of omics data (Dasgupta and De 2023). This integration allows for a more comprehensive understanding of the complex biological processes underlying leafy head development. Apart from that, ML techniques can handle the noise inherent in large-scale biological data (Libbrecht & Noble, 2015) while traditional statistical methods often struggle with the curse of dimensionality, where the number of features (e.g., genes) greatly exceeds the number of samples. In summary, ML provides a powerful framework for analyzing and integrating large-scale omics data to study complex traits like leafy head formation in *Brassica* species. GRNs also play a crucial role in understanding complex biological processes such as leafy head formation from large-scale datasets. A GRN represents the intricate net of interactions between genes, capturing the dynamic nature of gene expression and providing a systems-level view of how genes influence each other's activity (Springer et al. 2019; Chen et al. 2023; Huo et al. 2024). By integrating GRN analysis with ML approaches, researchers can uncover hidden patterns and relationships in large-scale transcriptomic data, leading to a more comprehensive understanding of the genetic mechanisms underlying complex traits (Yuan et al. 2024).

In our previous work, we successfully applied ML techniques to predict leaf adaxial-abaxial polarity genes in *Arabidopsis* using transcriptome and ChIP-seq data (Sun et al. 2024). This study demonstrated the power of ML in identifying novel genes associated with specific plant developmental processes and provided valuable insights into the genetic regulation of leaf polarity establishment. In the current study, we apply ML and GRN analysis to further understand the genetic basis of leafy head formation in *B. rapa* and* B. oleracea*. We chose these two species for parallel analysis based on three considerations: (1) it allows for the comparative analysis of gene expression patterns and regulatory pathways, enabling the identification of conserved mechanisms underlying leafy head formation; (2) doing analysis in parallel increases the robustness of our findings, as the consistency of results across the two species strengthens the confidence in the identified genes and regulatory networks; and (3) contrasting the genes between leafy head morphotypes of *B. rapa* and *B. oleracea* can provide insights into the evolutionary processes that have contributed to the morphotype diversity within the *Brassica* genus. Our specific objectives were to: (i) develop Random Forest models to predict novel heading-related genes using transcriptome data; (ii) construct GRNs to reveal the interactions among predicted genes; (iii) integrate ML predictions with network analysis to identify key regulatory modules; and (iv) perform comparative analysis to distinguish conserved- from species-specific regulatory patterns. Our integrative approach provides novel insights into the genetic architecture of leafy head formation and establishes a framework for accelerating crop improvement in *Brassica* species.

## Results

**Fig. 1:**
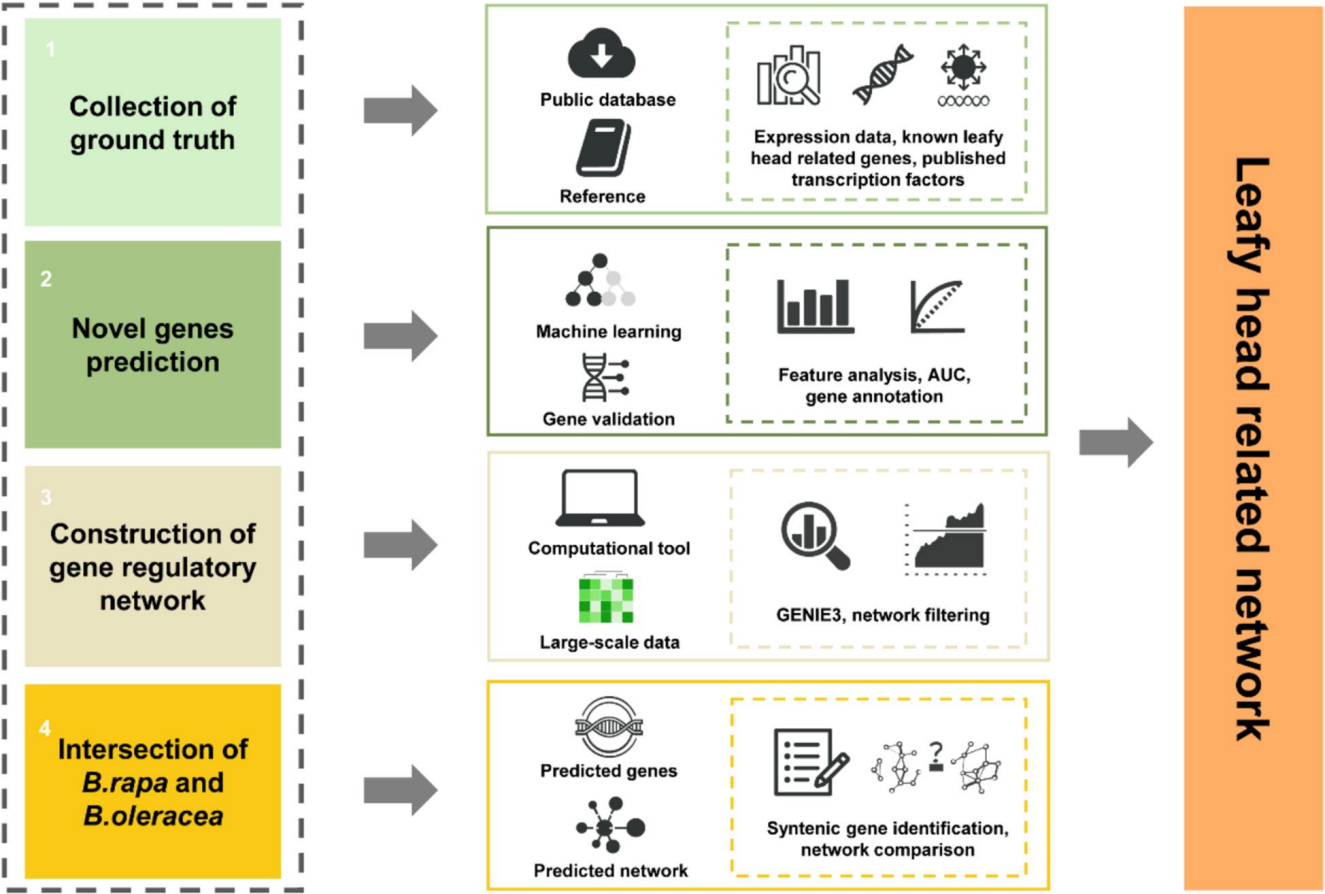
Workflow of approach to predict leafy head related genes and gene regulatory network. Four steps in this workflow include (i) collection of ground truth (genes, expression data) to use as input for ML and network prediction; (ii) prediction of novel genes related to leafy head formation using a Random Forest model; (iii) construction of GRNs between transcription factors and genes; and, (iv) integrative comparison between *B. rapa* and *B. olearcea*

To uncover the regulatory network of leafy head formation in *B. rapa* and *B. olearcea*, we employed an integrated approach combining ML predictions and comparative analysis between the two species (Fig. 1). The research workflow consisted of four key steps: (i) Collection of a knowledge base to use as input for ML and network prediction; (ii) Identification of novel genes involved in leafy head regulation using a Random Forest model; (iii) Construction of GRNs based on the genes predicted in step (ii). This step involved using the ML-predicted genes as seed nodes to build expanded TF networks, incorporating known regulatory interactions and potential new connections inferred from co-expression data and motif analysis; (iv) Comparative analysis of GRNs between *B. rapa* and *B. oleracea*. This final step included identifying conserved network modules, species-specific regulatory patterns, and key hub genes that may play central roles in leafy head formation across both species.

### Model construction for predicting genes involved in leafy head formation in *B. rapa* and *B. oleracea*

**Fig. 2:**
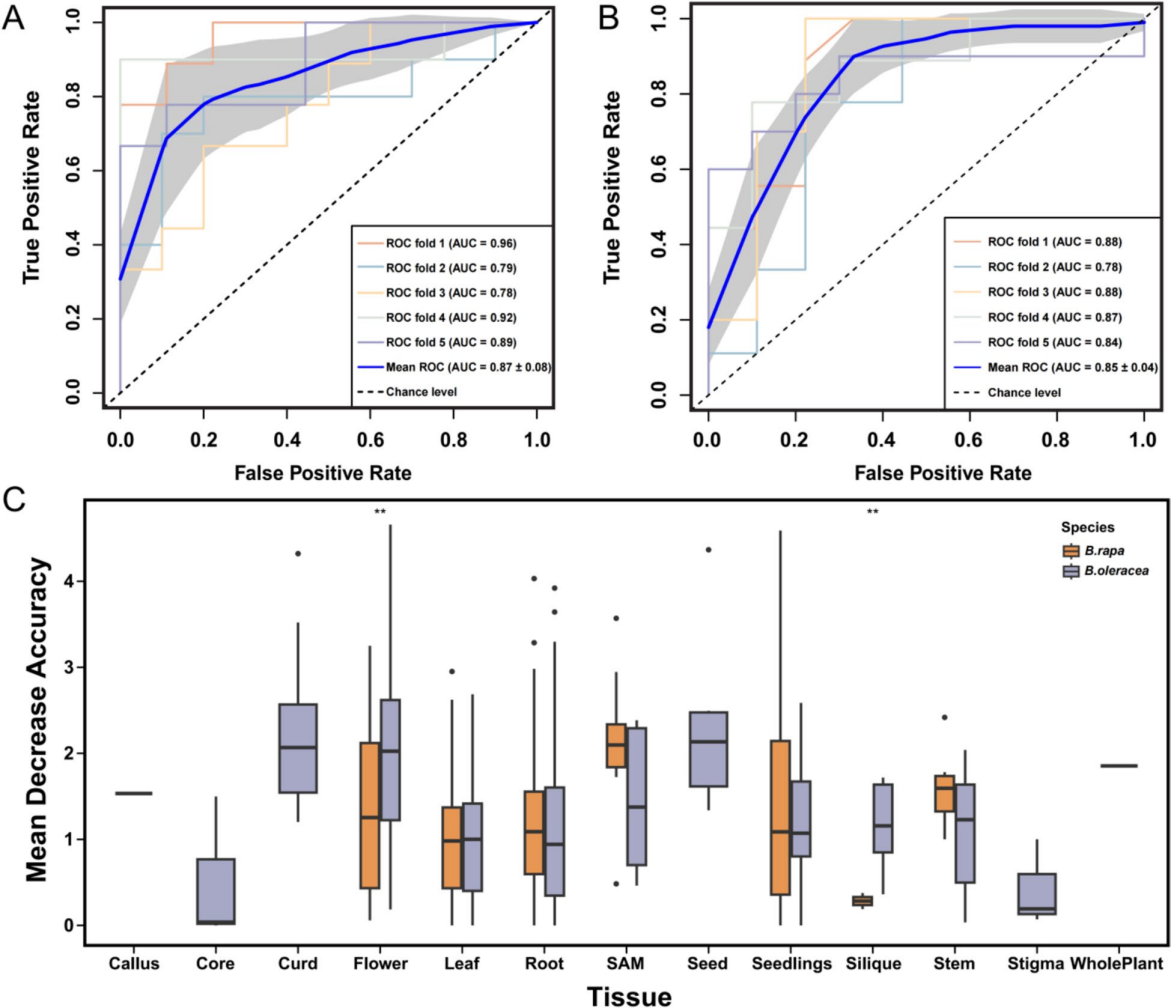
Performance evaluation and feature importance analysis of Random Forest models for predicting genes involved in leafy head formation in *B. rapa* and *B. oleracea*. **A** Receiver Operating Characteristic (ROC) curve with variability based on fivefold cross validation of *B. rapa* model. **B** ROC curve with variability based on fivefold cross validation of *B. oleracea* model. The solid blue line represents the mean ROC curve, the shaded grey area indicates the standard deviation of the ROC curves obtained from the 5 individual folds. The dashed diagonal line denotes the chance level, corresponding to an AUC of 0.5. **C** Comparison of feature importance scores across different tissues in *B. rapa* and *B. oleracea* models. The x-axis included the tissues which are present in one or two species. The y-axis represents the Mean Decrease Accuracy (MDA), with higher values indicating a greater contribution to the model's predictive performance. The color of each box indicates the species, with orange indicating *B. rapa* and purple indicating *B. oleracea*. Statistical significance of differences in MDA values between *B. rapa* and *B. oleracea* for each tissue was determined using a t-test, and significant differences (p < 0.05) are indicated by asterisks above the boxes

To develop the ML models, we utilized 634 and 762 normalized expression datasets (Supplementary Table 1) from *B. rapa* and *B. oleracea*, respectively, as input features. Among the *B. rapa* features, 444 were from heading morphotypes, while 190 were from non-heading morphotypes. In *B. oleracea*, 364 expression features were from heading morphotypes, and 398 features were from non-heading morphotypes. A ground truth set was then established which consisted of 47 well-characterized genes known to be involved in leafy head formation, designated as CLASS H (Heading) genes for both *B. rapa* and *B. oleracea* models (Supplementary Table 2). These genes were selected based on their reported functions in various aspects of leafy head formation, including polarity specification of the adaxial/abaxial axis, control of leaf curvature, meristem maintenance, small RNA pathway, modulation of hormone signaling pathways, and alteration of leaf morphology. While the CLASS H genes were identified based on available experimental information, there was a lack of ground knowledge for negative examples (i.e., genes not involved in leafy head formation). The random selection of “CLASS NH” genes as the negative set may lead to the disparity in expression levels between the positive and negative gene sets (Supplementary Fig. 1). To solve this issue and ensure a balanced training dataset, we employed a strategy to select an equal number of genes as CLASS NH (Non-Heading) genes (see details in Methods). These CLASS NH genes were chosen based on their overall similar expression levels to the CLASS H genes across various tissues and developmental stages. For both *B. rapa* and *B. oleracea* a Random Forest model was then developed independently, utilizing transcriptome raw datasets of heading and non-heading morphotypes. To assess the reliability of our models, we employed fivefold cross-validation to ensure that the models' performance was consistent across different subsets of the data. The *B. rapa* model achieved a mean Area Under the Curve (AUC) of 0.87 ± 0.08 (Fig. 2A), while the *B. oleracea* model demonstrated a mean AUC of 0.85 ± 0.04 (Fig. 2B). These high AUC values indicate that both models exhibit strong predictive capabilities in identifying genes associated with leafy head formation.

In order to investigate the importance of features, we use mean decrease accuracy (MDA), which quantifies each feature's contribution to the model's predictive performance, with higher values indicating greater importance. To examine the tissue-specific differences in feature importance between *B. rapa* and *B. oleracea*, we included all available tissues for each species: callus, core, curd, flower, leaf, root, SAM (shoot apical meristem), seed, seedlings, silique, stem, and whole plant. Features with a MDA greater than zero were used for comparison. It is important to note that some tissues were only present in one species due to either the presence of the specific organ or the availability of expression data. Boxplots were used to visualize the distribution of MDA values for each tissue in both species (Fig. 2C). Statistical analysis revealed significant differences in feature importance between *B. rapa* and *B. oleracea* for shared tissues. The MDA values for flower and silique tissues were significantly different between the two species (p < 0.05, t-test), suggesting that these tissues may play distinct roles during reproductive transitions of *B. rapa* and *B. oleracea*. In contrast, no significant differences were observed for the leaf, root, SAM, and seedlings tissues, indicating that these tissues may have similar importance in predicting heading-related genes in both species. Notably, curd tissue, which is unique to certain *B. oleracea* cultivars such as cauliflower and broccoli, showed high feature importance in the *B. oleracea* model. To further investigate the relationship between gene expression in specific tissues and the prediction of genes, we conducted partial dependence plot (PDP) analysis for the top 10 root and top 10 leaf tissues in both *B. oleracea* and *B. rapa*. The analysis of root tissues showed that 19 out of 20 examined root tissues from two species showed a negative impact on the prediction of genes. In contrast, the PDP analysis for leaf tissues did not reveal a clear, consistent pattern across the examined samples. The relationships between leaf tissue expression and predicted probability were more variable, with some tissues showing positive associations, and others negative effects. (Supplementary Fig. 2).

### Identification and functional analysis of predicted genes involved in leafy head formation

**Fig. 3:**
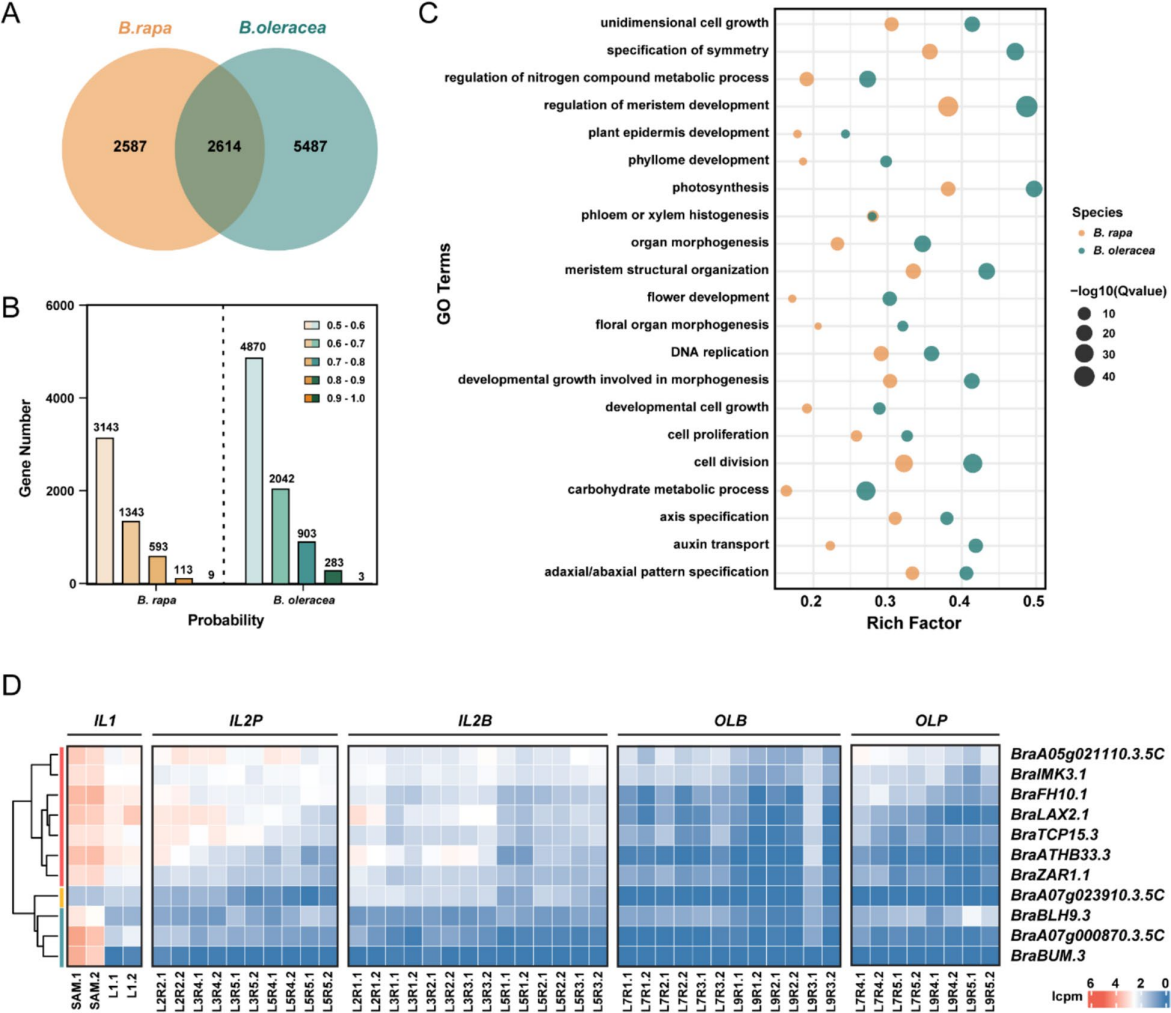
Functional analysis of genes predicted to be involved in leafy head formation (“preGenes”). **A** Venn diagram of preGenes between *B. rapa* and *B. oleracea,* the comparison was based on syntenic gene pairs. **B** Probability distribution of all preGenes in *B. rapa* and *B. oleracea* models, indicating how likely genes are according to the model to be involved in leafy head formation. **C** GO enrichment of preGenes shared between *B. rapa* and *B. oleracea* models. Richfactor for each GO term indicates the ratio between the gene numbers of preGenes and the total gene numbers of all annotated genes; dot size represents -log10 (Qvalue), for the significance of the enrichment; dot color represents the species (*B. rapa* in orange and *B. oleracea* in green). **D** Expression heatmap of 11 preGenes with high probability in *B. rapa* at the heading stage from Guo’s datasets. The color scale represents log-CPM (Counts Per Million) values. Tissue samples are labeled according to their spatial location, from inner (SAM) to outer leaves. IL1: inner leaves 1; IL2P: petiole of inner leaves 2; IL2B: blade of inner leaves 2; OLB: outer leaf blade; OLP: outer leaf petiole

After training the models, they were subsequently applied to all genes in both *B. rapa* and *B. oleracea* in order to predict novel genes potentially related to leafy head formation. Our final models predicted a substantial number of such genes potentially involved in leafy head formation (hereafter referred to as "preGenes" for predicted candidate genes) in *B. rapa* and *B. oleracea*. In *B. rapa*, 5,201 genes were identified as preGenes with a probability score greater than 0.5, while in *B. oleracea*, 8,101 preGenes were predicted with a probability above 0.5. To assess the robustness of these predictions, we performed sensitivity analysis on the ntree parameter, comparing our chosen setting (ntree = 500) with alternative values (ntree = 300 and ntree = 1000). This analysis revealed high consistency across different parameter settings, with over 94% overlap in gene identification for both species (Supplementary Fig. 3), confirming that our predictions are not artifacts of specific parameter choices. In order to compare these two sets of predictions, we focused on syntenic genes, which are orthologous genes located in similar positions on the genomes of different species, often sharing similar functions. By examining the syntenic relationships between the preGenes in *B. rapa* and *B. oleracea*, we found that 2,614 syntenic gene pairs were predicted as potential candidates in both species, with probability scores above 0.5 (Fig. 3A). The distribution of preGenes probabilities in *B. rapa* and *B. oleracea* revealed a positive correlation between the two species, with a substantial number of gene pairs displaying similar predicted probabilities to be involved in heading in both models (Supplementary Fig. 4, r = 0.54, p < 0.001).

Among all preGenes (syntenic or non-syntenic), 9 genes in *B. rapa* and 3 genes in *B. oleracea* exhibited exceptionally high probability scores greater than 0.9, representing 11 unique genes in total as *TCP15.3* appeared in both species, indicating their strong potential involvement in leafy head development (Fig. 3B, Supplementary Table 1). We prioritized genes with probability scores > 0.9 based on multiple considerations: (1) these represent the top 0.17% (9/5201) and 0.04% (3/8101) of predictions in *B. rapa* and *B. oleracea* respectively, suggesting high confidence; (2) this stringent threshold reduces false positives while identifying the most promising candidates; (3) the presence of TCP15.3 with identical high scores (0.91) in both species provides internal validation of our approach. In order to further interpret the predictions, Gene Ontology (GO) enrichment was performed on all the preGenes from *B. rapa* and *B. oleracea* models (Fig. 3C; Supplementary Table 3, 4). We chose to focus on the shared GO terms between these two species, as both are known to form leafy heads despite their evolutionary divergence. The analysis revealed many significantly enriched GO terms common to both *B. rapa* and *B. oleracea*, including unidimensional cell growth (GO:0009826), adaxial/abaxial pattern specification (GO:0009955), auxin transport (GO:0060918) and regulation of meristem development (GO:0048509). These shared terms likely represent key molecular pathways and mechanisms underlying leafy head formation.

To further validate our predictions, we examined the expression patterns of the 11 highly predicted genes shared between *B. rapa* and *B. oleracea* using a dataset derived from Guo’s study on Chinese cabbage (*B. rapa*) at the heading stage, providing transcriptome profiles of 24 spatially dissected tissues representing different regions from inner to outer leaves of a mature chinese cabbage plant. The heatmap reveals three distinct expression profiles for these genes: the first group, including *TCP15.3, IMK3.1, FH10.1, LAX2.1, ATHB33.3, ZAR1.1*, and *BraA05g021110.3.5C* shows a clear gradient of expression across the leaf tissues. These genes exhibit high expression in the inner leaves (IL1) and gradually decrease in expression from the inner to outer leaves. The second group, comprising *BUM.3, BLH9.3*, and *BraA07g000870.3.5C*, displays a preferential expression in the SAM. The third group consists of a single gene, *BraA07g023910.3.5C*, which exhibits low expression levels across all leaf tissues examined (Fig. 3D). To validate the robustness of our prediction methodology, we performed leave-one-out cross-validation using all 47 known leafy head formation genes. This validation demonstrated robust performance with 39/47 genes (83.0%) and 36/47 genes (76.6%) successfully predicted (probability ≥ 0.5) in B. rapa and B. oleracea respectively, both significantly exceeding random expectation (hypergeometric test, P < 0.001). Key regulatory genes showed consistently high prediction probabilities across both species, such as *PHB.1* (0.82 in both), *KNAT1* (0.792 and 0.842), and *YAB2.3* (0.838 and 0.844), confirming the reliability of our ML approach in dentifying leafy head formation genes (Supplementary Table S5).

### Construction of gene regulatory networks for transcription factors in *B. rapa* and *B. oleracea*

The ML approach identified many novel genes potentially involved in leafy head formation. However, the regulation of complex traits like leafy head formation is likely to involve complex GRNs rather than individual genes. To investigate the regulatory landscape of transcription factors (TFs) in *B. rapa* and *B. oleracea*, we constructed comprehensive GRNs. These GRNs were based on the same transcriptome datasets that were utilized as input features in the ML models. Initially, 2,840 and 2,918 TFs were identified in *B. rapa* and *B. oleracea*, respectively, from the plantTFDB database. Transcriptome datasets from various tissues and treatments were used to infer putative TF-target interactions, with a threshold applied to edge weight importance score of 0.01. This led to 332,429 interactions in *B. rapa* and 382,103 interactions in *B. oleracea*. To explore the modular structure of these extensive networks, we applied the iGraph partitioning algorithm, which revealed the presence of 10 sub-clusters in *B. rapa* and 9 sub-clusters *B. oleracea*. These sub-clusters represent clusters of genes with highly interconnected regulatory relationships, providing a biologically meaningful representation of the complex GRNs.

### Integration of machine learning predictions and gene regulatory networks

**Fig. 4:**
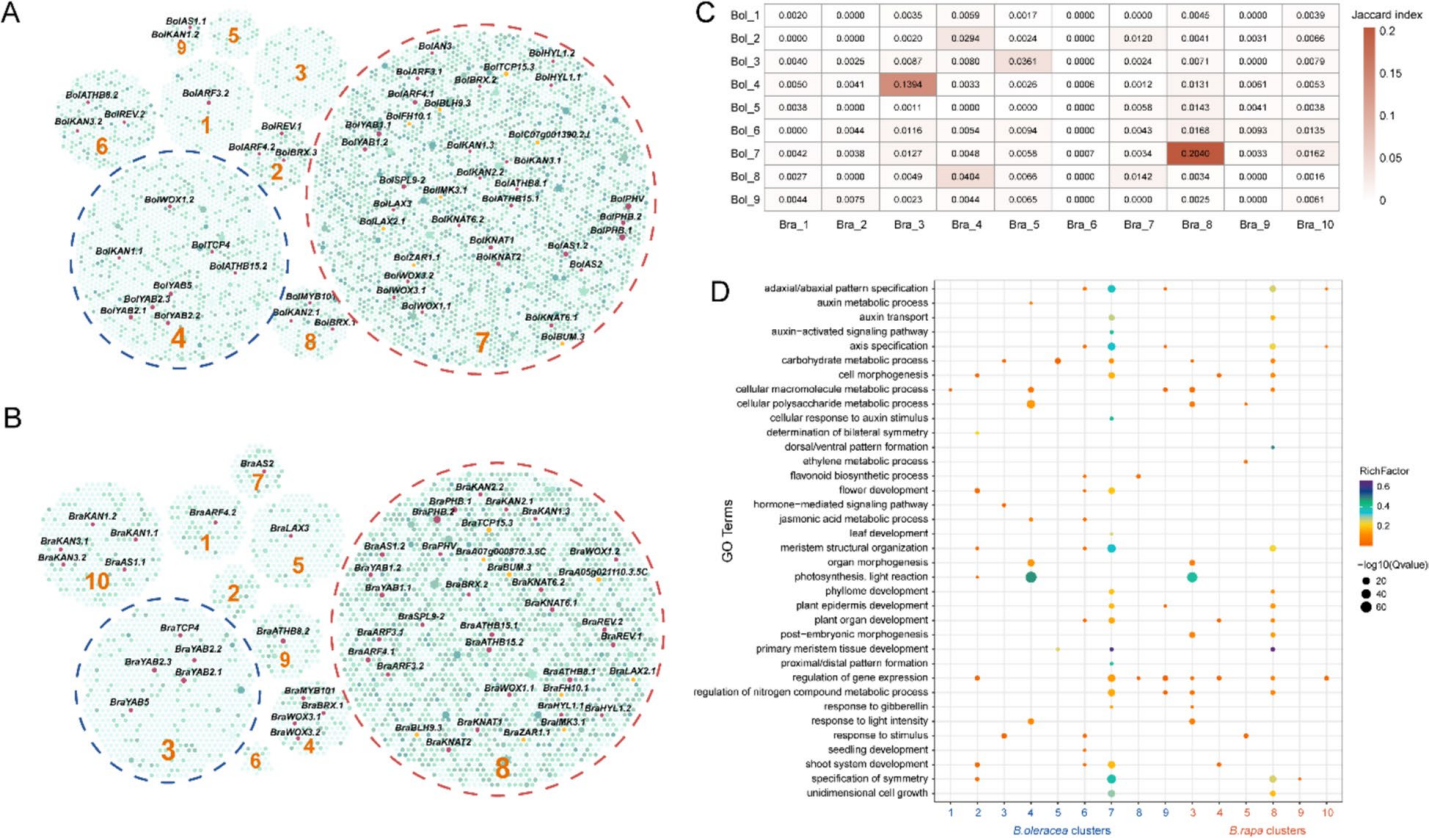
Comparative analysis of predicted genes (preGenes) in the gene regulatory networks (GRNs) of *B. rapa* and *B. oleracea*. **A** *B. oleracea* GRN: The GRN is divided into 9 sub-clusters, with nodes representing genes and edges representing regulatory interactions. Known ground truth genes (47 genes) are colored in red, while preGenes are colored in green. The high probabilitypreGenes from Table 1 are colored in yellow. **B** *B. rapa* GRN: The GRN is divided into 10 sub-clusters, with the same color scheme. **C** Heatmap of syntenic gene distribution of preGenes between *B. rapa* and *B. oleracea* clusters. The color intensity represents the Jaccard index of syntenic genes shared between each pair of clusters, with deep red indicating a higher number of shared genes. **D** GO enrichment analysis of preGenes in each cluster of *B.rapa* and *B. oleracea* GRNs. Only clusters with statistically significant terms are displayed. The dot size represents the -log10(Qvalue), with larger dots indicating a higher significance of the enrichment. The color of each dot represents the rich factor, which indicates the ratio between the number of preGenes and the total number of annotated genes in each GO term

To gain insights into the regulatory network of the preGenes involved in leafy head formation, we intersected the preGenes with the constructed TF GRNs for *B. rapa* and *B. oleracea*. From the *B. rapa* model, 4,856 from the total 5,201 preGenes (473 of which are TFs, Supplementary Table 6) were found in the *B. rapa* TF GRNs while 7,597 from the total 8,101 preGenes (724 of which are TFs, Supplementary Table 7) were found in *B. oleracea*. To further elucidate the regulatory relationships among preGenes, we extracted the interactions specific to the preGenes from the larger TF GRNs. This process generated a subset of the regulatory network that focuses on the preGenes. After the extraction process, 18,792 interactions were retained in the *B. rapa* network (Supplementary Table 8), while 44,139 interactions remained in the *B. oleracea* network (Supplementary Table 9). To visualize and analyze the preGene regulatory networks, we assigned the preGenes to the previously identified sub-clusters. The resulting preGene regulatory networks for *B. rapa* and *B. oleracea* showcase the intricate interactions among the preGenes and their associated TFs. In the GRN of *B. oleracea* (Fig. 4A), preGenes were found to be distributed across all nine sub-clusters, indicating their widespread involvement in various regulatory modules. To gain a deeper understanding of the interactions involving key genes, we examined the distribution of known ground truth genes within the network. Notably, *B. oleracea* cluster 7 was found to be the sub-cluster with the largest number of known ground truth genes, with a total of 27. Interestingly, when we investigated the distribution of the 12 preGenes with high probability identified in Table 1, we discovered that all of them were distributed within cluster 7. Similarly, in the GRN of *B. rapa* (Fig. 4B), the preGenes were found to be distributed across all 10 sub-networks. Upon examining the distribution of known genes, we found that cluster 8 contained the highest number, with a total of 27 known genes. Remarkably, the 12 highly preGenes from Table 1 were also located within cluster 8. The concentration of heading-related genes in *B. oleracea* cluster 7 and *B. rapa* cluster 8 reveals these as core regulatory modules for leafy head formation. Based on the known functions of genes within these two clusters, we observed enrichment for three key processes essential for heading: (1) leaf polarity establishment (containing *YAB1, AS1, PHB* genes), which determines the adaxial-abaxial axis crucial for leaf curvature; (2) meristem regulation (containing *KNAT1*, *KNAT2* genes), controlling the balance between stem cell maintenance and organ initiation; and (3) auxin-mediated growth (containing auxin response genes *ARF3*, *ARF4*), coordinating differential cell expansion. This enrichment of heading-related processes strongly indicates the important role of cluster 7 and cluster 8 in leafy head formation.

**Table 1:** PreGenes with high probability in *B. rapa* and *B. oleracea* models

Gene_name	Probs_of_bra	Probs_of_bol
*LAX2.1*	0.928	0.840
*BUM.3*	0.918	0.890
*BraA07g000870.3.5C*	0.914	0.628
*FH10.1*	0.912	0.816
*IMK3.1*	0.912	0.840
*TCP15.3*	0.910	0.910
*ZAR1.1*	0.908	0.702
BraA05g021110.3.5C^*^	0.906	
*BLH9.3*	0.904	0.796
*BolC06g023810.2 J*	0.764	0.904
*ATHB33.3*	0.816	0.902

^*^BraA05g021110.3.5C only have one prob since it doesn’t have a syntenic gene in *B.olearcea*

### Comparative analysis of heading network in *B. rapa* and *B. oleracea*

To elucidate the evolutionary conservation and divergence of leafy head formation mechanisms between *B. rapa* and *B. oleracea*, we performed a comparative analysis based on the Jaccard index of preGenes in their respective GRNs. The Jaccard index matrix reveals several instances of conserved gene distribution between the two species, as well as some notable divergences (Fig. 4C). The highest Jaccard index (0.20) is observed between *B. oleracea* cluster 7 and *B. rapa* cluster 8, indicating a substantial overlap in gene content and suggesting functional conservation between these clusters. Notably, both clusters contain numerous known CLASSH genes, including *YAB1.1, YAB1.2, AS1.2, PHB.1, PHB.2, KNAT6.1*, and *KNAT6.2*, further supporting their functional importance in leafy head formation. Additionally, *B. oleracea* cluster 4 shows relatively high similarity with *B. rapa* cluster 3, with a Jaccard index of 0.14. The presence of shared ground truth genes such as *YAB2.1, YAB2.2, YAB2.3, YAB5*, and *TCP4* in both clusters further indicates the functional conservation and important role in leafy head formation of two species.

The GO enrichment analysis of preGenes in various groups of *B. oleracea* and *B. rapa* Gene GRNs reveals high similarities, with many terms appearing in both (Fig. 4D). Many leafy head formation-related terms such as adaxial/abaxial pattern specification (GO:0009955), axis specification (GO:0009798), plant epidermis development (GO:0090558) and auxin transport (GO:0060918) were highly enriched in both *B. oleracea* cluster 7 and *B. rapa* cluster 8, suggesting these two clusters play crucial roles in leafy head formation. Apart from that, some other clusters also showed similarities in different terms. For instance, *B. oleracea* cluster 4 and *B. rapa* cluster 3 were significantly enriched in photosynthesis light reaction (GO:0019684) and light intensity (GO:0009642), indicating a focus on energy production. Multiple clusters, including *B. oleracea* cluster3, *B. oleracea* cluster5, *B. oleracea* cluster7, *B. rapa* cluster 3, and *B. rapa* cluster 8 were significantly enriched in carbohydrate metabolic processes (GO:0005975), indicating its critical role for leafy head formation.

**Fig. 5:**
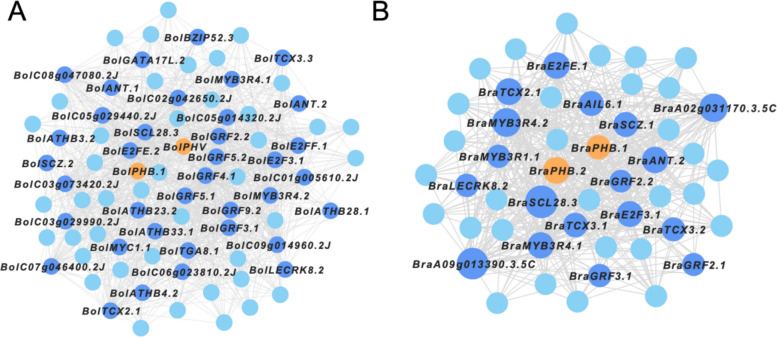
Core regulatory sub-networks of leafy head formation in (**A**) *B. oleracea* and (**B**) *B. rapa*. Nodes in the networks represent genes, with orange nodes indicating known ground truth genes involved in leafy head formation, deep blue nodes representing other transcription factors (TFs), and light blue nodes representing other genes. Edges between nodes signify regulatory interactions between genes

In order to gain deeper insights into the regulatory mechanisms underlying leafy head formation, we focused on two key sub-GRN clusters: cluster 7 in *B. oleracea* and cluster 8 in *B. rapa*. These clusters exhibited the highest Jaccard index (0.20), indicating substantial gene content overlap, and were enriched for many leafy head formation-related GO terms. To further identify the core genes within the key sub-GRNs of *B. rapa* and *B. oleracea*, we performed a network centrality analysis. Four centrality measures (betweenness, closeness, degree, and eigenvector) were calculated for each gene in the sub-GRNs to capture different aspects of a gene's importance within the network. To ensure robustness, we used a combination of all four centrality measures for screening core genes (see Methods for detailed descriptions). Applying this strict selection criterion, we obtained a set of core genes that consistently exhibited high centrality values. In *B. oleracea* cluster 7 (Fig. 5A), 81 core genes were finally identified, including 39 TFs such as *BolANT.1, BolANT.2, BolGRF5.1*, and *BolGRF9.2*. In *B. rapa* cluster 8 (Fig. 5B), a total of 50 core genes were identified, including 20 TFs such as *BraGRF2.1*, *BraGRF2.2*, *BraAIL6.1* and *BraSCL28.3*. Interestingly, some core genes, such as *BolPHB.1*, *BolPHV*, *BolANT.2, BolGRF2.2* and *BolGRF3.1*, have syntenic genes in *B. rapa* cluster 8 (*BraPHB.1*, *BraPHB.2*, *BolANT.2, BraGRN2.2* and *BraGRF3.1*, respectively), suggesting a conserved role in leafy head formation.

## Discussion

Our machine learning approach successfully identified a substantial number of novel genes potentially involved in leafy head formation in both *B. rapa* and *B. oleracea*. The selection of a proper training set is a critical step in ML, as it directly impacts the model's performance and the biological relevance of its predictions. We developed a strategy to select negative genes based on their similar expression levels to positive genes across various tissues and developmental stages. This matching strategy minimizes potential biases in the training data, ensuring the model captures true biological differences between positive and negative genes rather than simply distinguishing between high and low expression levels. This approach increases the likelihood of identifying novel genes truly associated with leafy head formation.

By comparative analysis of feature importance between *B. rapa* and *B. oleracea*, we identified an interesting pattern of the tissue-specific contributions to the prediction of genes involved in leafy head formation. The lack of significant differences in feature importance for leaf and seedlings tissues suggests that they may share common regulatory pathways or gene expression patterns related to leafy head formation in *B. rapa* and *B. oleracea.* This observation is consistent with previous studies that have highlighted the involvement of leaf and seedling tissues in the regulation of leafy head formation (Burian et al. 2022; Gu et al. 2017; Husbands et al. 2015; Mao et al. 2014). Similarly, the root tissues also do not differ significantly between the two species. The PDP analysis of the top root tissues in both species showed a consistent negative impact on the prediction of genes. This pattern aligns with the expectation that genes crucial for head development would be preferentially expressed in aerial tissues, rather than in roots. In contrast, the variable feature importance observed from PDP analysis of leaf tissues underscores the complex genetic regulation underlying leafy head development, like diverse spatial and temporal regulation of gene expression across different leaf regions and developmental stages. Future research should focus on integrating data from various leaf tissues, developmental stages, and experimental conditions to build a more complete picture of the genetic mechanisms in *Brassica* crops. Functional enrichment analysis of preGenes revealed conserved mechanisms governing leafy head formation in *Brassica* species. Several pathways known to be crucial for leafy head formation and morphogenesis were significantly enriched in both species, reinforcing their importance in the heading process. The enrichment of meristem development terms aligns with observations that leafy heads and curds in *Brassica* species typically have larger meristems compared to non-heading morphotypes, contributing to the formation of compact, tightly packed structures. These findings expand our understanding of the molecular basis of leafy head formation.

Our computational approach identified 11 genes potentially involved in leafy head formation in *Brassica* crops. To gain further insights into their potential functions, we analyzed their expression profiles using the spatially dissected tissue dataset from Guo et al. (2022a, b). This analysis revealed diverse expression patterns among the 11 genes, ranging from high expression in inner leaves with decreasing expression towards outer leaves, preferential expression in the SAM, and consistent low expression across all leaf tissues. Interestingly, these diverse expression patterns were found to be consistent with the biological functions of the genes. For example, *BUM* (also called *STM*), a Class I knotted-like homeodomain protein required for SAM formation and maintenance in *Arabidopsis*, showed preferential expression in the SAM. This expression pattern aligns with its role in preventing the incorporation of meristem center cells into differentiating organ primordia, which is crucial for maintaining the integrity of the SAM and regulating leaf initiation during leafy head formation (Balkunde et al. 2017). Similarly, *TCP15*, a gene involved in the regulation of cell expansion in *Arabidopsis* leaves (Rath et al. 2022), also showed higher expression in the SAM and petiole of inner leaves. This expression pattern supports its potential role in promoting cell expansion and division during the early stages of leafy head formation, contributing to the morphology of leaves in the head (Alemán-Báez et al. 2024; Liu et al. 2024). Additionally, *TCP15* has been shown to be required for elongation and gene expression responses to auxin (Ferrero et al. 2021). Given the crucial role of auxin in leaf development and morphogenesis, the higher expression of *TCP15* in the SAM and petiole of inner leaves suggests that it may mediate auxin-dependent processes, such as cell elongation and differentiation, which are essential for shaping the leaves and maintaining the structural integrity of the leafy head (Guo et al. 2022b; Alemán-Báez et al. 2024; Liu et al. 2024). Furthermore, Guo's gene expression analysis revealed 9,133 differentially expressed genes (DEGs) across leaves of the mature leafy head, which were grouped into 14 clusters. The patterns of the 11 genes corresponded to their first 3 clusters, characterized by the highest expression in the youngest leaves. The cluster with preferential expression in the SAM in the study of Guo is the only cluster enriched for the GO term ad/abaxial axis specification, to which many of our class H genes belong. This analysis not only served as an independent validation of our predictions but also offered valuable insights into the spatial and temporal dynamics of gene expression during the complex process of leafy head formation.

Gene regulatory network construction allowed us to uncover complex regulatory relationships among preGenes. By integrating ML predictions with these networks, we identified key regulatory clusters driving leafy head formation. Specific sub-clusters containing high numbers of known ground truth genes and preGenes suggest their important roles in heading regulatory networks. Comparative analysis revealed several instances of conserved gene distribution across clusters between species, indicating these clusters may represent core regulatory modules preserved since evolutionary divergence. The presence of ground truth genes in conserved clusters further supports their critical roles. Species-specific GO enrichments in hormone-related processes suggest regulatory divergence between *B. rapa* and *B. oleracea*. For example, the ethylene metabolic process was enriched only in *B. rapa*, aligning with Zhang et al. (2022a, b), who demonstrated the ethylene pathway as a specific artificial selection target in Chinese cabbage (*B. rapa*) but not in cabbage (*B. oleracea*). They showed ethylene treatment induced increased leaf angle and more compact architecture in Chinese cabbage, while cabbage showed no response and formed heads without ethylene pathway upregulation. Additional species-specific enrichments in flavonoid biosynthesis and jasmonic acid metabolism further highlight regulatory divergences. These findings illuminate both shared developmental pathways and species-specific adaptations contributing to distinct characteristics in leafy head formation.

The identification of core genes within the largest key sub-GRNs of *B. rapa* and *B. oleracea* through network centrality analysis provides further insights into the key regulators of leafy head formation. Some TFs such as *GRF2, GRF3, ANT,* and *TCX3* were identified in both *B. rapa* and *B. oleracea* clusters, suggesting a conserved role for these genes in the regulation of leafy head formation across species. It has been found that *GRF2* and *GRF3* act as positive regulators of plant growth: *BrGRF*-overexpressing *Arabidopsis thaliana* plants developed larger leaves, flowers as well as longer roots than the wild type (Hong et al. 2017). In the context of leafy head formation, *GRF2* and *GRF3* may contribute to the development of leafy heads by promoting cell proliferation and organ growth since the rapid growth and expansion of leaves are necessary for the formation of tightly packed and overlapping leaf structures characteristic of leafy heads (Mao et al. 2014). This is further supported by Alemán-Báez (2024) who identified a role for miR396b-5p targeting *Arabidopsis thaliana* orthologues of *GRF3* in pointed cabbage leafy head formation, highlighting the importance of GRF family genes in leafy head formation. *ANT* is usually expressed in the lateral shoot organ primordia and floral organ primordia and is required for the control of cell proliferation (Mizukami and Fischer 2000). Loss of function alleles have abnormal ovules and abnormal lateral organs in *Arabidopsis* (Elliott et al. 1996). Moreover, *ANT* modulates auxin biosynthesis via the regulation of *YUC4* (Y. J. Li et al. 2021a, b, c), indicating its involvement in the auxin signaling pathway, which is crucial for leafy head formation. This regulation likely contributes to strong apical dominance in plants, suppressing side shoot formation and promoting the development of a compact, tightly wrapped head structure. *TCX3* is expressed in stamens and immature ovules, and it’s involved in cell fate determination and cell division processes (Simmons et al. 2019). The conservation of *TCX3* in the key sub-GRNs of both *B. rapa* and *B. oleracea* suggests a potential role in the regulation of leafy head development. The precise regulation of cell division and differentiation is crucial for the initiation of leafy head development, where differential growth along the leaf blade is essential to initiate leaf curvature followed by growth of tightly packed leaves (Liu et al. 2024; Van Lijsebettens and Clarke 1998), and *TCX3* may play a role in coordinating these processes during leafy head formation. Apart from the conserved core genes, we also identified genes that play roles in only one species network. For example, *GRF5*, which was found in the *B. oleracea* key sub-GRN network, has been shown to cause an increase in leaf size when overexpressed in *Arabidopsis* (35S:GRF5, Beltramino et al. 2018). Larger leaves with increased surface area may contribute to the formation of rosette leaves, that support the growth of the leafy head, but can also contribute to the formation of more compact and tightly packed leaf structures, enhancing the overall size and shape of the leafy head. These findings provide valuable insights into the conservation and divergence of regulatory mechanisms between *B. rapa* and *B. oleracea* and will facilitate the development of targeted breeding strategies and the identification of key genes for leafy head formation.

Our study provides a comprehensive framework for understanding the complex genetic architecture of leafy head formation. The integrated ML and network analysis approach identified novel candidate genes, expanding our understanding of this trait. The parallel detection of similar genes and networks in both species provides mutual validation, reinforcing the robustness of our findings and highlighting the conservation of key regulatory mechanisms. While experimental validation in *Brassica* remains challenging due to genome complexity and transformation difficulties, our computational predictions provide valuable candidates for future functional characterization and targeted breeding efforts to improve leafy head traits in *Brassica* crops. Moreover, our integrative approach demonstrates the potential of combining computational predictions with network analysis to accelerate gene discovery and elucidate complex processes in plants.

## Method

### Expression data sources

Multiple raw datasets across different biological conditions and tissues were downloaded from the SRA repository of the National Center for Biotechnology Information (Supplementary Table 1). Trimming was applied using Trimmomatic v.0.38 (Bolger et al. 2014) to remove sequence artifacts, including adapter contamination and low-quality nucleotides. Using STAR (Dobin et al. 2013) with default stringencies and parameters, post-trimming reads of *B. rapa* were aligned to the Chiifu 3.5 genome (Z. Zhang et al. 2022a, b) and post-trimming reads of *B. oleracea* were aligned to the JZS 2.0 genome (Cai et al. 2020). We mapped the read loci to RNA features using the featureCounts function of Subread (Liao et al. 2014). This resulted in 634 expression features of *B. rapa* and 762 expression features of *B. oleracea*. To normalize the data and account for differences in library size across samples, we transformed the raw count data into Log Counts Per Million (LCPM) values using edgeR (Robinson et al. 2010). LCPM normalization is a widely used method for RNA-seq data normalization that takes into account the varying sequencing depths of different samples. By transforming the raw counts into LCPM values, we effectively standardize the expression data, making it comparable across different samples and tissue types.

### Machine learning classification

The *B. rapa* randomforest model was built based on the above defined 634 expression features using the randomForest R package (Liaw and Wiener 2002). The number of trees was set to 500 and mtry (the number of features to consider at each split point) was set to 25. The *B. oleracea* randomforest model was built based on the 762 expression features using the same package, the number of trees was set to 500 and mtry was set to 27.

A total of 47 genes related to leafy head formation reported in the literature were treated as “CLASS H” genes (Supplementary Table 2, Machida et al. 2015; Liang et al. 2016; Zhang et al. 2021; P. Li et al. 2021a, b, c; Tabusam et al. 2023). Given that there is no definite knowledge of which genes are not involved in leafy head formation, we developed a method to select a set of “CLASS NH” genes that exhibited similar expression distributions to the “CLASS H” genes. First, we calculated the median expression value for each gene in the dataset. Next, we created bins of these median values, with each bin containing 100 genes. For each “CLASS H” gene, we randomly selected a “CLASS NH” gene from the same bin, ensuring that the selected “CLASS NH” gene had a very similar median expression to its corresponding “CLASS H” gene. This approach allowed us to obtain a balanced set of “CLASS NH” genes that closely matched the expression distribution of the “CLASS H” genes. In this way, the genes were divided into a modeling set (i.e., 47 “CLASS H” genes and the 47 “CLASS NH” genes) and a prediction set (the remaining genes). Five-fold cross validation was performed to evaluate the performance of the model. This process divided the modeling set into 5 equal subsets, where 4 subsets were used for training and 1 for validation in each fold. Importantly, each gene was evaluated exactly once as part of validation data and was never used simultaneously for both training and validation within the same fold. The CLASS H probability produced by the model was used as the prediction score; genes with probability greater than 0.5 were classified as novel “CLASS H” genes. The Receiver Operating Characteristic curve (ROC curve) and the Area Under the ROC Curve (AUC) were computed using the R package ROCR (Sing et al. 2005) for the evaluation of the model performance. The Mean decrease accuracy was computed using the importance function from the R package Randomforest (Liaw and Wiener 2002).

### Construction of gene regulatory network of *B. rapa* and *B. oleracea*

The TFs of *B. rapa* and *B. oleracea* were both collected from PlantTFDB (Jin et al. 2017). The expression matrix from the “Expression data sources” section and TF list were then used to identify coexpression interactions between TFs and putative target genes. For the GRN construction, we first implemented appropriate data normalization procedures. The expression data was processed by log-CPM (Counts Per Million) transformation using the edgeR package (Robinson et al. 2010) to account for differences in sequencing depth across samples, resulting in the normalized expression matrix used as input for our analysis. GENIE3 was used to construct the GRN (Huynh-Thu et al. 2010), which utilizes feature selection with tree-based ensemble methods. For network inference, we used the following specific parameter settings in GENIE3: Random Forest (RF) was selected as the tree method, the number of candidate regulators randomly selected at each tree node was set to the square root of the total number of regulators, and 50 trees were used per ensemble. Each interaction in the GENIE3 network is assigned a score based on its weight relative to the maximum weight of all interactions. A weight cutoff of 0.01 was chosen to filter out less significant interactions. To assess the robustness of our findings, we also performed a sensitivity analysis using a more permissive threshold of 0.005, which confirmed the stability of the key regulatory modules (see Results). R-iGraph package (Csardi G & Nepusz T, 2006) was used for clustering the interactions with a resolution of 0.5; this package implements a multi-level modularity optimization algorithm for finding community structure (Blondel et al. 2008). The final network was visualized by Gephi (version 0.10.1, Bastian et al. 2009).

### Comparative analysis of GRN between *B.rapa* and *B. oleracea*

Syntenic gene pairs were identified by SynOrths (Cheng et al. 2012) to investigate the evolutionary conservation and divergence of GRNs between *B. rapa* and *B. oleracea*. The pheatmap R package (Raivo Kolde 2010) was used to visualize the distribution of syntenic preGenes across the GRN clusters of *B. rapa* and *B. oleracea*. The Jaccard index was used to quantify the overlap of preGenes between GRN clusters across the two species, calculated as the ratio of shared preGenes to the total number of unique preGenes in both clusters. This measure ranges from 0 (no shared genes) to 1 (identical gene sets), allowing us to identify evolutionarily conserved regulatory modules between *B. rapa* and *B. oleracea*. Higher Jaccard indices indicate greater conservation of gene content between clusters, suggesting functional similarity in their regulatory roles during leafy head formation. To further characterize the functional roles of the preGenes in each GRN cluster, we performed GO enrichment analysis using the clusterProfiler (Yu et al. 2012) package in R. To identify the core genes within the sub-GRNs of *B. rapa* and *B. oleracea*, a network centrality analysis was performed using the CytoNCA plug-in of Cytoscape software (Tang et al. 2015). Four centrality measures (betweenness, closeness, degree, and eigenvector) were calculated for each gene in the sub-GRNs. Betweenness centrality quantifies how often a node lies on the shortest paths between other nodes, reflecting its role in connecting different parts of the network (Freeman 1977). Closeness centrality measures the average shortest path length from a node to all other nodes, indicating how quickly information can spread from that node to the rest of the network (Beauchamp 1965). Degree centrality is the number of edges connected to a node, representing its direct influence on the network (Rodrigues 2019). Eigenvector centrality assigns higher scores to nodes connected to other high-scoring nodes, capturing the importance of a node based on the importance of its neighbors (Bonacich 1972). These centrality measures have been widely used to identify key players in biological networks (Ma et al. 2014; Mishra et al. 2022; Han et al. 2023; Zhu et al. 2023). To ensure robustness, we used a combination of all four centrality measures for screening core genes. The median values of these four measures were determined separately, and a gene was considered a candidate core gene if all four of its centrality measures were greater than their respective median values. To further refine the core gene set and reduce the final number of genes, this process was repeated three times for each species, retaining only those genes that consistently met the criteria across all iterations. By using this strict selection approach, we were able to identify the most central and influential genes within the sub-GRNs that potentially play crucial roles in the regulation of leafy head formation.

## Supplementary Information

Supplementary Material 1.

Supplementary Material 2. Fig. S1 Comparison of gene expression patterns between randomly selected “CLASS NH” genes and “CLASS NH” genes selected based on expression levels. The rows represent individual genes, while the columns represent expression data with different tissue samples or experimental conditions. The color scale indicates the level of gene expression, with red representing high expression, blue representing low expression, and white representing intermediate levels. The above heatmap is based on random selected “CLASS NH” genes as the negative set which display a notable disparity in expression levels between the positive (red) and negative (green) gene sets. The bottom heatmap was made based on “CLASS NH” genes selected with expression levels comparable to the positive genes which display a more balanced expression levels between the positive and negative gene sets. Fig. S2 Partial Dependence Plots (PDPs) for top 10 root and leaf tissues of *B. oleracea* and *B. rapa *models. Fig. S3 Sensitivity analysis of Random Forest models to the ntree parameter in *B. rapa *and *B. oleracea* models. (A) Venn diagrams showing the overlap of genes identified by Random Forest models with different ntree settings in *B. rapa *(left) and *B. oleracea* (right) models. (B) Probability distributions of unique genes from different ntree settings in *B. rapa* (left) and *B. oleracea* (right) models. Fig. S4 Comparison of syntenic gene pair probabilities (p < 0.001).

## Data Availability

All relevant data are presented in the online Supplementary Materials.

## Abbreviations

ML: Machine Learning
GRN: Gene Regulatory Network
ROC: Receiver Operating Characteristic
MDA: Mean Decrease Accuracy
AUC: Area Under the Curve
PDP: Partial Dependence Plot
GO: Gene Ontology
TFs: Transcription Factors
DEGs: Differentially Expressed Genes
