# Supplementary material for: Integrative machine learning and gene regulatory network analysis identifies novel genes for leafy head formation in Brassica rapa and B. oleracea
Source: Mol Hortic. 2026 Jun 8;6:45. doi: 10.1186/s43897-026-00230-1 (PMC13244942; doi:10.1186/s43897-026-00230-1)
Supplement: Supplementary file 2 — Supplementary Material 2. Fig. S1 Comparison of gene expression patterns between randomly selected “CLASS NH” genes and “CLASS NH” genes selected based on expression levels. The rows represent individual genes, while the columns represent expression data with different tissue samples or experimental conditions. The color scale indicates the level of gene expression, with red representing high expression, blue representing low expression, and white representing intermediate levels. The above heatmap is based on random selected “CLASS NH” genes as the negative set which display a notable disparity in expression levels between the positive (red) and negative (green) gene sets. The bottom heatmap was made based on “CLASS NH” genes selected with expression levels comparable to the positive genes which display a more balanced expression levels between the positive and negative gene sets. Fig. S2 Partial Dependence Plots (PDPs) for top 10 root and leaf tissues of B. oleracea and B. rapa models. Fig. S3 Sensitivity analysis of Random Forest models to the ntree parameter in B. rapa and B. oleracea models. (A) Venn diagrams showing the overlap of genes identified by Random Forest models with different ntree settings in B. rapa (left) and B. oleracea (right) models. (B) Probability distributions of unique genes from different ntree settings in B. rapa (left) and B. oleracea (right) models. Fig. S4 Comparison of syntenic gene pair probabilities (p < 0.001). [file 43897_2026_230_MOESM2_ESM.docx]

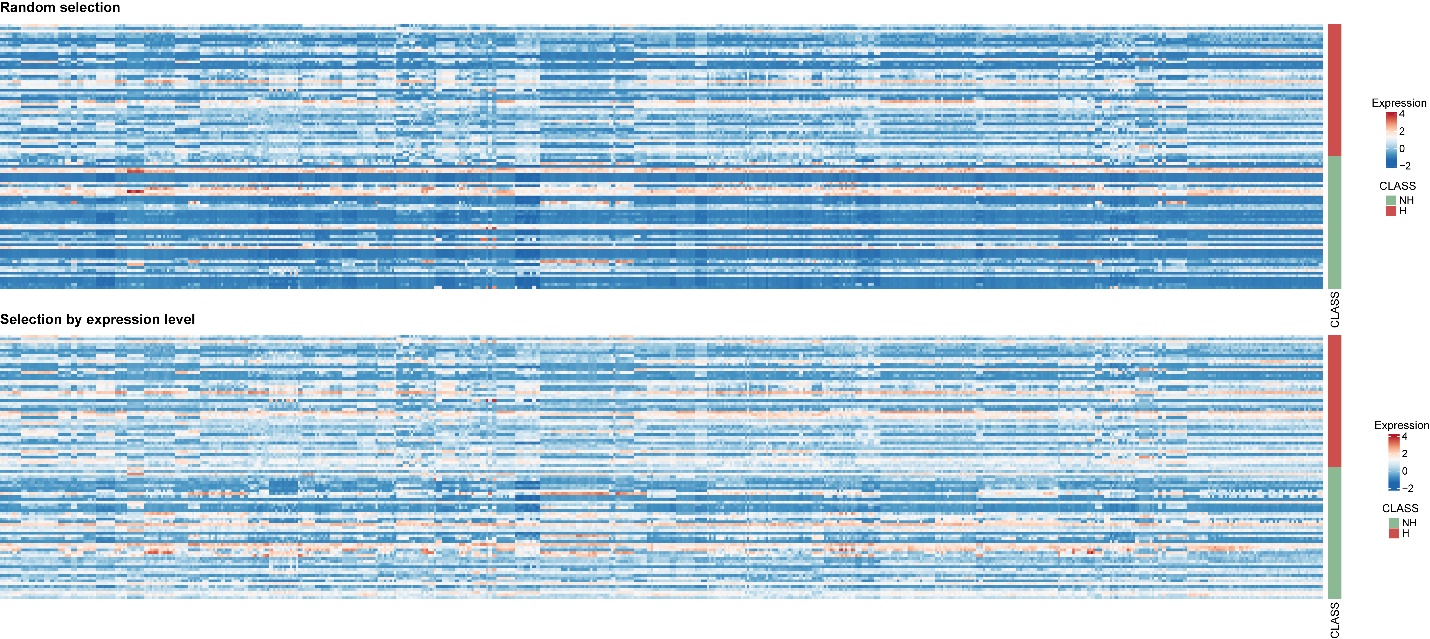


**Supplementary Fig. 1** Comparison of gene expression patterns between randomly selected “CLASS NH” genes and “CLASS NH” genes selected based on expression levels. The rows represent individual genes, while the columns represent expression data with different tissue samples or experimental conditions. The color scale indicates the level of gene expression, with red representing high expression, blue representing low expression, and white representing intermediate levels. The above heatmap is based on random selected “CLASS NH” genes as the negative set which display a notable disparity in expression levels between the positive (red) and negative (green) gene sets. The bottom heatmap was made based on “CLASS NH” genes selected with expression levels comparable to the positive genes which display a more balanced expression levels between the positive and negative gene sets.


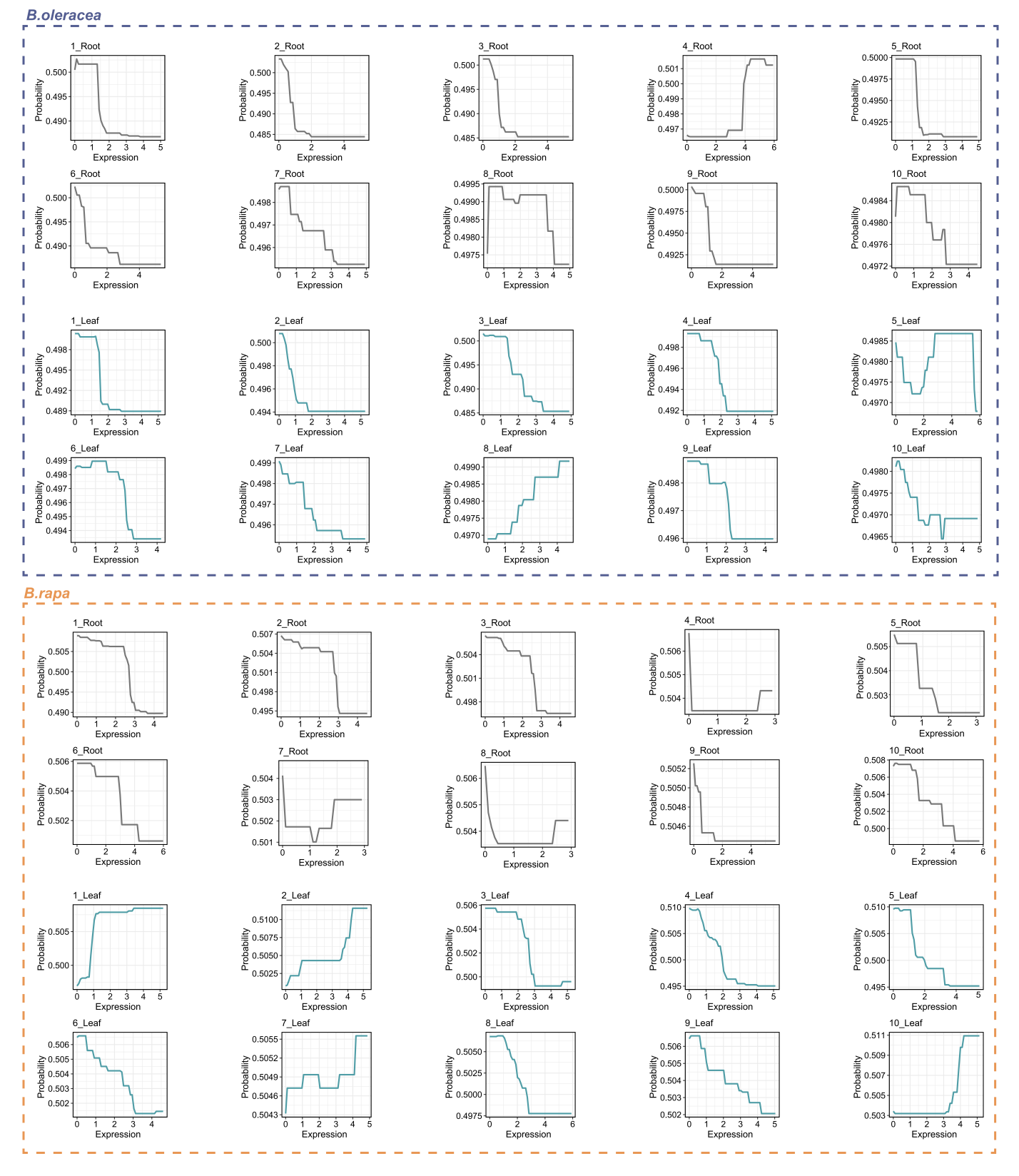


**Supplementary Fig. 2** Partial Dependence Plots (PDPs) for top 10 root and leaf tissues of *B. oleracea* and *B. rapa* models.


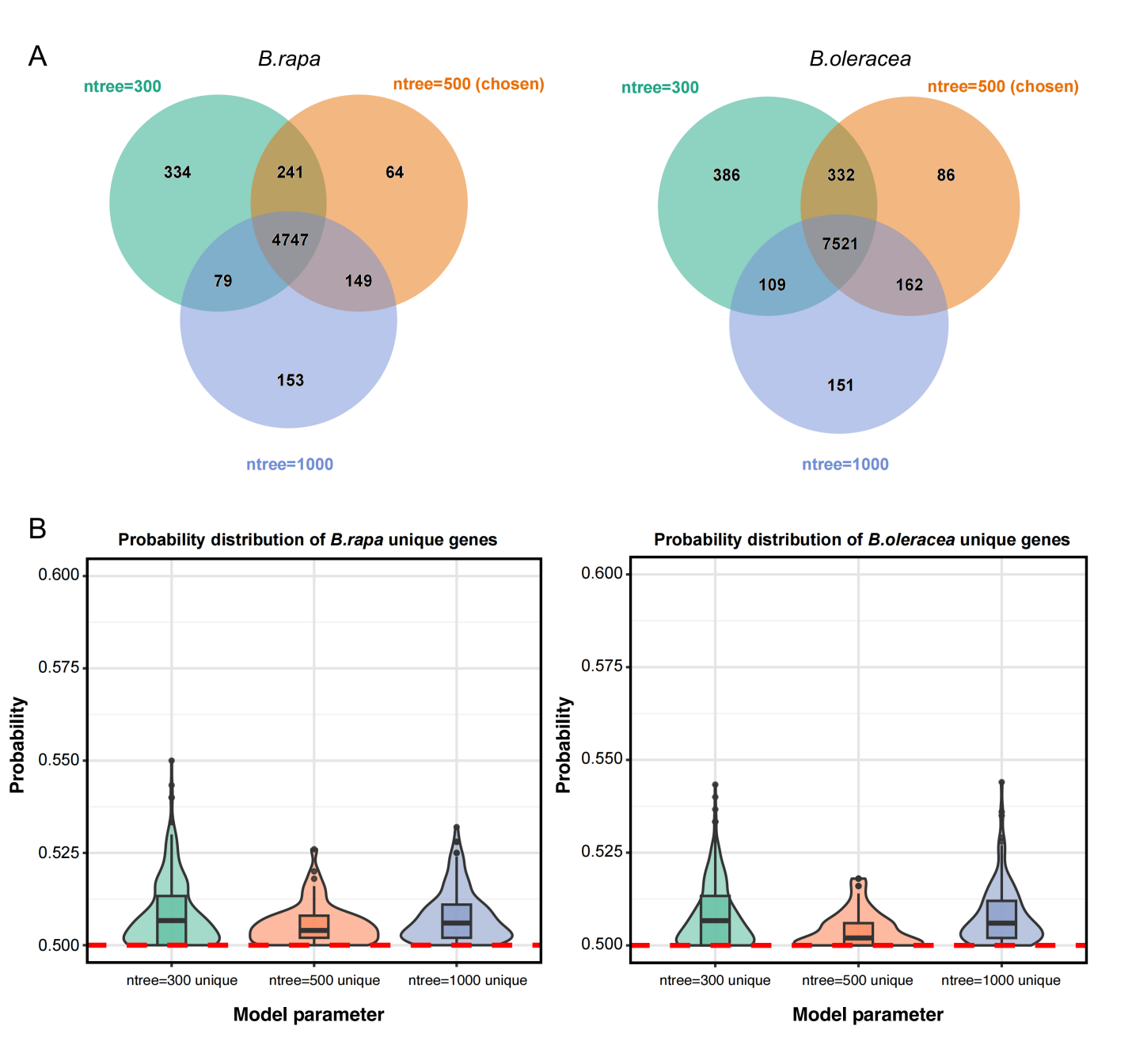


**Supplementary Fig. 3** Sensitivity analysis of Random Forest models to the ntree parameter in *B. rapa* and *B. oleracea* models. (A) Venn diagrams showing the overlap of genes identified by Random Forest models with different ntree settings in *B. rapa* (left) and *B. oleracea* (right) models. (B) Probability distributions of unique genes from different ntree settings in *B. rapa* (left) and *B. oleracea* (right) models.


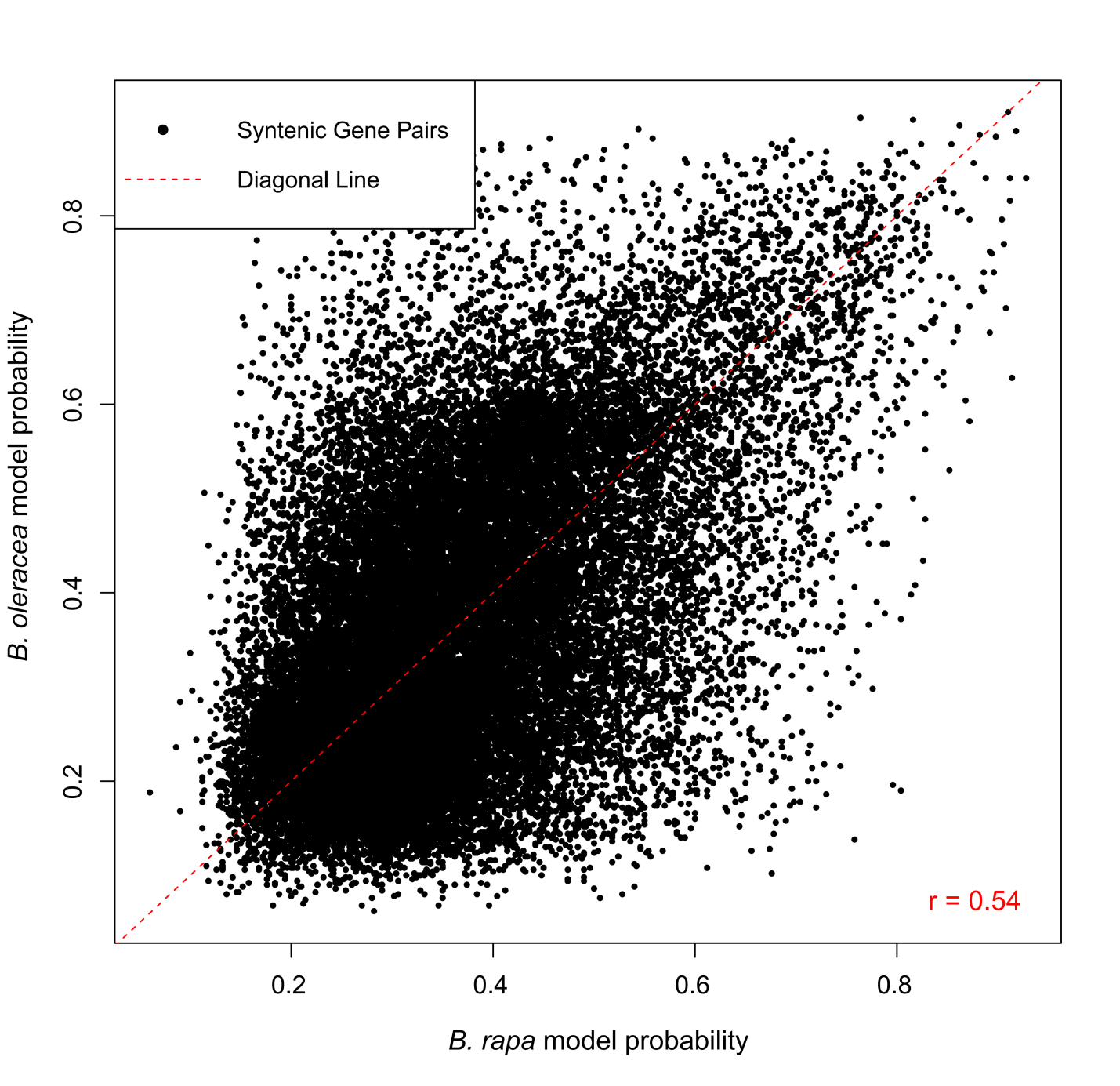


**Supplementary Fig. 4** Comparison of syntenic gene pair probabilities (p < 0.001).
